# Characterization of *Escherichia coli* and other bacteria isolated from condemned broilers at a Danish abattoir

**DOI:** 10.3389/fmicb.2022.1020586

**Published:** 2022-11-10

**Authors:** Ahmed Alfifi, Jens P. Christensen, Yaovi Mahuton Gildas Hounmanou, Marianne Sandberg, Anders Dalsgaard

**Affiliations:** ^1^Department of Veterinary and Animal Sciences, Faculty of Health and Medical Science, University of Copenhagen, Frederiksberg, Denmark; ^2^Department of Veterinary Public Health, College of Veterinary Medicine, King Faisal University, Al-Ahsa, Saudi Arabia; ^3^National Food Institute, Danish Technical University, Lyngby, Denmark

**Keywords:** food safety, cellulitis, systemic infection, meat inspection, hepatitis, *E*. *coli*

## Abstract

Meat inspection is important to ensure food safety and protect public health. Visual inspection of slaughtered carcasses for pathological changes should be supported by bacteriological analysis to determine whether the entire carcass or parts of it should be condemned. The aim of this study was to determine the bacterial species present in different sample types from condemned broiler carcasses. Furthermore, we investigated the genetic characteristics, zoonotic potential, and relatedness of *Escherichia coli*, the predominant bacterial species isolated from the carcasses. A total of 400 broiler carcasses condemned because of cellulitis (100), scratches (100), hepatitis (100), and healthy control carcasses (100) were selected. Samples of meat, pathological lesion, and bone marrow of each carcass were obtained for microbial analysis. From the analyzed samples, 469 bacterial isolates were recovered with *E*. *coli* accounting for 45.8%, followed by *Aeromonas* spp. (27.9%), in particular *A*. *veronii*. The highest rate of bacterial isolation was observed in carcasses condemned with cellulitis, whereas carcasses with hepatitis had the lowest rate of bacterial isolation. Forty-four *E*. *coli* isolates originating from different sample types were selected for whole genome sequencing. A clonal relationship was shown between *E*. *coli* from different sample types of the same carcass condemned with cellulitis and scratches. A major clade of *E*. *coli* was found in carcasses condemned with cellulitis with isolates containing *mdf*(A), *tet*(A), and *bla*_TEM-1B_ genes that confer resistance to macrolides, tetracycline, and ampicillin, respectively. *E*. *coli* in this clade all belonged to ST117 and clustered with *E*. *coli* isolates previously collected from dead chickens and carcasses condemned due to cellulitis in Denmark, Finland, and the United Kingdom. Bacterial evaluation results of carcasses condemned with cellulitis, scratches (moderate to severe skin lesion), and acute hepatitis confirmed the need for total condemnation of carcasses with these pathological findings. A similar evaluation should be done for carcasses affected with chronic hepatitis, and minor scratches lesions.

## Introduction

Poultry meat inspection is primarily conducted to ensure that safe and wholesome meat is produced for human consumption. Data obtained from poultry meat inspection are also used for the surveillance of animal welfare conditions and animal diseases (EFSA, 2012; Arzoomand et al., 2019). Meat inspection is regulated by EU legislation that ensures that official inspections are performed in a uniform way. The inspection of broilers in the slaughterhouse includes evaluations of health conditions on-farm before slaughter as well as ante-mortem and post-mortem inspection (Törmä et al., 2021).

In Denmark, the Danish Veterinary and Food Administration is responsible for performing meat inspection in the abattoir, and to record inspection data. The meat inspection data is entered into the Danish Quality Assurance System (KIK) database which is managed by the broiler industry and also contains other data from breeding to retail for all individual broiler flocks. A recent study by Alfifi et al. (2020) showed that 1.1% of broiler carcasses in Denmark were condemned at post-mortem inspection. Globally, the condemnation rate has been estimated to be 1.04%, with cellulitis accounting for 14.2% of the condemned carcasses (Salines et al., 2017; Bernd et al., 2020). Other studies have confirmed that cellulitis is one of the main causes of carcass condemnation at slaughter (Kumor et al., 1998; Gomis et al., 2000; Derakhshanfar and Ghanbarpour, 2002). In 2018, cellulitis accounted for 25% of all condemnations in Germany (Bernd et al., 2020). In Denmark, the most frequently registered cause of condemnation was skin lesions (scratches and dermatitis/cellulitis) representing more than 50% of the condemnation causes (Alfifi et al., 2020). Cellulitis is characterized by the presence of yellow skin discoloration in the area that surrounds the cloacae (Poulsen et al., 2018; Bernd et al., 2020) indicating inflammation underneath the skin, which can be seen as pus and/or fibrin. Cellulitis is usually not considered to result in clinical disease or to affect the growth performance of birds (Elfadil et al., 1996). Most often it is unnoticed during rearing and consequently leads to condemnation of carcasses when detected at the slaughterhouse (Poulsen et al., 2018).

Scratches are seen during post-mortem inspection as an injury that usually occurs at the caudal part of the limb. Findings such as scratches, as well as injuries, hematomas, footpad dermatitis, breast blisters, and hock burn provide further information on welfare conditions during rearing, transportation, and handling of broilers (Allain et al., 2009; Gouveia et al., 2009; Iannetti et al., 2020). A positive relationship between the prevalence of scratches and systemic infection was recently reported in a slaughtered flock of broilers (Alfifi et al., 2020). However, there seems little if any information available about levels of systemic infection in carcasses with scratches at the individual bird level.

Findings of acute hepatitis will result in condemnation of the carcass and the organs (Ministry of Environment and Food of Denmark, 2018). Acute hepatitis is regarded as a sign of a systemic infection whereas chronic hepatitis is characterized by localized pathological changes in the liver. Consequently, if a disease process results in systemic infection, the entire carcass should be condemned whereas chronic pathological changes may only require condemnation of the affected part of the carcass (Danish Meat inspection circular, 2005; Herenda, 2019). Post-mortem meat inspection of chickens is traditionally based on visual observations of lesions, which is subjective and sometimes not a true reflection of the actual infection status of the carcass (Alfifi et al., 2020).

*Escherichia coli* is the most common cause of bacterial disease in poultry resulting in significant health problems and economic losses (Nolan et al., 2013). Colibacillosis is characterized by different clinical and pathological manifestations, including septicemia, airsacculitis, peritonitis, salpingitis, and omphalitis (Nolan et al., 2013; Poulsen et al., 2017). Chickens may be a source of extra-intestinal pathogenic *E*. *coli (*ExPEC) (Johnson Murray et al., 2003; Johnson et al., 2008, 2009; Bonnet et al., 2009; Lyhs et al., 2012; Johnson et al., 2017). Phylogenetic and clonal relatedness between ExPEC strains from humans and animals indicate that such strains share virulence-associated genotypic or phenotypic traits (Rodriguez-Siek et al., 2005; Moulin-Schouleur et al., 2007; Johnson et al., 2008). ExPEC strains from chickens can also cause a diverse range of extra-intestinal infections in animal models typical for putative ExPEC pathotypes (Stromberg et al., 2017; Mellata et al., 2018) Thus, poultry may be a source of ExPEC strains with zoonotic potential and therefore pose a potential threat to food safety and human, but also animal health (Meena et al., 2020). Moreover, broilers may be a significant source of multidrug-resistant *E*. *coli* which represents a serious public health risk (Falgenhauer et al., 2019).

Condemnations by food inspections at slaughterhouses account for the second largest proportion (19.3%, 17 million tonnes) of the total food waste generated in the EU (FUSIONS, 2016). In Denmark, the annual wastage resulting from condemnation of broiler carcasses is estimated to be about 1,300 tonnes of meat (Alfifi et al., 2020). Reducing food wastage is essential to ensure food security, maximize commercial returns for food producers, and minimize the environmental impacts of food production activities and food consumption. Thus, there is a need to conduct research to determine if broiler carcasses currently condemned as unsuitable for human consumption represent actual health risks.

Therefore, the overall aim of our study was to provide knowledge about the bacterial flora and food safety aspects of broiler carcasses condemned at abattoirs. We characterized the bacteria and possible systemic infections associated with the most common causes of condemnation. Antimicrobial resistance and genetic characteristics including possession of virulence genes in *E*. *coli* commonly isolated from pathological lesions of condemned carcasses were determined as was the genetic relatedness of the *E*. *coli* isolated from the meat, lesions, and bone marrow of individual carcasses.

## Materials and methods

### Sample collection

A total of 400 condemned and healthy carcasses were collected during five monthly visits to a main slaughterhouse in Denmark from October 2020 to February 2021. Three hundred carcasses condemned due to cellulitis, scratches, and acute hepatitis were obtained, 100 of each. In addition, 100 healthy carcasses were randomly selected. Carcasses condemned due to cellulitis and scratches were collected from the slaughter line after the defeathering stage, while healthy carcasses, and carcasses condemned with hepatitis were collected after the evisceration stage where the pluck was removed. The broiler carcasses originated from 28 different poultry farms representing 43 flocks and 41 houses.

### Carcass collection and handling

Carcasses were selected from the slaughter line immediately after being condemned by meat inspection of the plucks and placed individually in a sterile plastic bag that was labeled as “cellulitis,” “scratches,” “hepatitis,” and “control.” Collected carcasses condemned with scratches represented carcasses with moderate to severe skin lesions. Whereas carcasses affected with hepatitis were selected from those with acute hepatitis. Immediately after collection, the carcasses were transferred and maintained at 4°C in a refrigerator at the slaughterhouse. The carcasses were subsequently transported in an insulated box with ice packs to a laboratory of the University of Copenhagen, Denmark. Upon arrival sampling processing and bacterial isolation was initiated within 48 h of carcass collection.

### Sample collection and bacterial isolation

From every condemned carcass three samples were obtained, one from the lesion, one from the meat (breast muscle), and one from the bone marrow of the femur. From each healthy carcass, samples were collected from meat, bone marrow, subcutaneously (control for cellulitis and scratches), and from the liver. A heated branding iron was used to sterilize the surfaces before sampling the breast muscle and liver. Subsequently, a sterile metal swab with cotton placed at the edge which was inserted into the sterilized tissue and streaked onto a 5% blood agar plate consisting of blood agar base (Oxoid, United Kingdom) and sterile bovine blood. Samples obtained from cellulitis and scratches were taken directly from the lesion. The outer surface of the inflamed skin of carcasses with cellulitis was aseptically incised carefully to access the lesion (fibrin/pus). Subsequently, the sterile metal swab was inserted in the lesion and then streaked onto a blood agar plate. Samples from scratches were carefully obtained from the deep lesion by cutting around the damaged skin. The sterile metal swab was placed in the lesion and then streaked onto a blood agar plate. Samples from healthy chickens were obtained from under the skin, by cutting gently the outer layer and inserting the sterile metal swab under the skin in a new area that had not been touched or exposed, and then streaked onto a blood agar plate. Samples from bone marrow were obtained after removing the muscle carefully around femur, then cutting the end of the femur with bone shears, and subsequently, the metal swab was carefully inserted directly into the bone marrow and streaked onto a blood agar plate. The bone shears was sterilized in 70% alcohol solution before and after use. Isolation of bacteria from the bone marrow was considered as a systemic infection. During the 5 months of sampling, data about the total number of slaughtered chickens approved for human consumption as well as condemned carcasses were obtained from the Danish Quality Assurance System (KIK) database.

The blood agar plates were incubated at 37°C under aerobic conditions for 24 to 48 h. From plates with growth, up to five colonies were sub-cultured to obtain pure cultures. On agar plates with colonies displaying different morphologies, one specimen of each colony type was obtained for pure culture and bacterial identification. Blood agar plates containing less than five colonies were discarded.

### Bacterial identification

Matrix-Assisted Laser Desorption Ionization Time-of-Flight Mass Spectrometry (MALDI-TOF MS) from VITEK® MS (bioMérieux, France) was used to confirm the species of the bacterial isolates.

### Statistical analyses

A Chi-square test was used to determine the significance of systemic infection occurrence (isolation of bacteria from bone marrow) in the three causes of condemnation (cellulitis, scratches, and hepatitis) when compared to control samples. A value of *p* was considered statistically significant if the value was ≤0.05.

### Antimicrobial susceptibility testing of *Escherichia coli*

Forty-four *E*. *coli* isolates originating from carcasses condemned with cellulitis (20 isolates), scratches (12 isolates), and hepatitis (12 isolates) was subjected to antimicrobial susceptibility analysis to the following antimicrobials: ampicillin (AMP) (dilution range 64–1 μg/mL), azithromycin (AZI) (64–2 μg/mL), cefotaxime (CTA) (4–0.25 μg/mL), ceftazidime (CTZ) (8–0.5 μg/mL), chloramphenicol (CHL) (128–8 μg/mL), ciprofloxacin (CIP) (8–0.015 μg/mL), colistin (COL) (16–1 μg/mL), gentamicin (GEN) (32–0.5 μg/mL), meropenem (MER) (16–0.03 μg/mL), nalidixic acid (NAL) (128–4 μg/mL), sulfamethoxazole (SME) (1024–8 μg/mL), tetracycline (TET) (64–2 μg/mL), tigecycline (TIG) (8–0.25 μg/mL), and trimethoprim (TRI) (32–0.25 μg/mL). The minimum inhibitory concentrations (MIC) were determined by the broth microdilution method according to ISO-standard (20776–1). *Escherichia coli* ATCC25922 was used as a control strain. Results interpretation was done using the Thermo Scientific™ Sensititre™ SWIN™ Software System. The breakpoints from EUCAST version 11.0 for Enterobacteriaceae were used.^1^

### Whole genome sequencing of *Escherichia coli*

The 44 *E*. *coli* isolates were cultured onto blood agar and incubated at 37°C for 24 h. A pure bacterial colony was then transferred to Luria broth (Difco, United States) and incubated in a shaking incubator at 37°C for 13 h. Genomic DNA was extracted using the bacterial DNA extraction kit Maxwell® RSC Cultured Cells DNA Kit (Promega, United States) following the manufacturer’s protocol. Quality control of the extracted DNA was assessed using NanoDrop 8,000, Qubit (Thermo Scientific, Waltham, MA, United States) and by gel electrophoresis in a 1% agarose gel stained with ethidium bromide. Whole genome sequencing was performed according to standard protocols with Illumina MiSeq (Illumina, San Diego California United States). The raw reads were submitted to NCBI GenBank Database under the project accession number PRJNA888805.

### Bioinformatic analysis

We used the following software for analyzing the data from the whole genome sequencing. The quality of raw reads was evaluated using FastQC version v0.11.9 (Andrews, 2010). Trimming raw sequences was done with Trimmomatic version 0.35. The genomes were assembled using Spades 3.9 (Bankevich et al., 2012). Quality control of the assembled genomes was accessed using QUAST version v5.0. *In silico* assembled sequences were analyzed using different tools from the servers of the Center for Genomic Epidemiology (CGE)^2^ for multi-locus sequence types using MLST 2.0 (Wirth et al., 2006; Jaureguy et al., 2008; Larsen et al., 2012), and antimicrobial resistance genes using ResFinder 4.1. (Camacho et al., 2009; Bortolaia et al., 2020). Virulence determinants were assessed in the genomes using VirulenceFinder 2.0. To detect plasmids, we used PlasmidFinder 2.1 with the Enterobacteriaceae database, with a 95% threshold for minimum identity and a minimum of 60% for coverage.

### Phylogenetic analysis

In the phylogenetic analysis, we wanted to determine the genetic relatedness of the isolated *E*. *coli* strains with other *E*. *coli* strains obtained elsewhere in Europe. Our 44 strains were therefore included in a phylogenetic analysis with 120 publicly available *E*. *coli* genomes. Seventy of these publicly available genomes were human isolates originating from Denmark, whereas 50 other genomes were isolates from poultry obtained in Denmark, Sweden, Finland, and the United Kingdom. Details and accession numbers of the public genomes included in the phylogenetic analysis are shown in (Supplementary Table S2).

Single-nucleotide variants (SNVs) were called with Snippy v4.6.0^3^ under the following parameters: mapping quality of 60, a minimum base quality of 13, minimum read coverage of 4, and a 75% concordance at a locus. An alignment of core genome SNVs was produced in Snippy v4.1.0 for phylogeny inference. Putative recombinogenic regions were detected and masked with Gubbins version v2.4.1(Croucher et al., 2015). A maximum likelihood (ML) phylogenetic tree was built, with RAxML version/8.2.12, under the GTR model with 100 bootstraps (Stamatakis, 2014). The final tree was rooted on the *E*. *coli* K12 genome and visualized with Microreact.

## Results and discussion

### Bacteria isolated from condemned carcasses

#### Post-mortem causes of condemnation

During the study period from October 2020 to February 2021, a total of 17,331,511 chickens were slaughtered at the slaughterhouse. A total of 17,125,632 carcasses were healthy and approved for human consumption during the post-mortem inspection, while 205,879 (1.2%) carcasses were condemned. The main causes of carcass condemnation include scratches (48,649), cellulitis (39,534), and hepatitis (17,217). All slaughtered flocks were free of *Salmonella* spp. as registered in the national surveillance program as according to Danish laws any flock positive for *Salmonella* spp. cannot be slaughtered in Denmark.

#### Isolated bacterial species

At two initial visits to the slaughterhouse, all samples were processed and agar plates incubated under both aerobic and anaerobic conditions. Both incubations yielded agar plates with bacterial colonies; however, when the identity of such colonies were confirmed by MALDI-TOF MS there were no differences in the bacterial species identified. We, therefore, decided only to culture agar plates under aerobically conditions in subsequent sampling. A total of 469 bacterial isolates were recovered from the 400 carcasses. *E*. *coli* was the most common bacterial species isolated from all sample types (45.8%; 215/469), followed by *Aeromonas* spp. (27.9%; 131/469). Among the isolated *Aeromonas* spp., *A*. *veronii* was predominant (71.8%; 94/131), followed by *A*. *sobria* (11.5%), *A*. *hydrophila* (10.7%), *A*. *caviae* (3.8%), *A*. *ichthiosmia* (1.5%), and *A*. *salmonicida* (0.8%).

#### Carcasses condemned with cellulitis

From the carcasses with cellulitis, bacteria were detected in 100% of the sampled lesions. Furthermore, bacteria were isolated in 48.0% of bone marrow and 12.0% of meat samples from these carcasses. *E*. *coli* (60.9%) was the most frequently isolated species from the carcasses with cellulitis followed by *Aeromonas* spp. (20.6%) and *P*. *mirabilis* (12.5%) (Table 1). The common isolation of *E*. *coli* is consistent with several reports among birds with cellulitis (Singer et al., 2000; Poulsen et al., 2018; Sarowska et al., 2019; Azam et al., 2020). Cellulitis usually develops from initial skin scratches, which allow entry of bacteria into the subcutaneous tissue where they subsequently proliferate and cause infection (Jeffrey et al., 2004; Bernd et al., 2020). However, as carcasses with cellulitis in the current study were free from scratches, the development of cellulitis may be attributed to other predisposing factors, i.e., wet irritated skin in combination with stress, genetic factors, nutritional deficiency, population density, litter quality, and breeders’ and hatchery hygiene (Dziva and Stevens, 2008; Allain et al., 2009; Kabir, 2009; Poulsen et al., 2018; Alfifi et al., 2020; Iannetti et al., 2020; van den Oever et al., 2020). A recent investigation of management factors associated with cellulitis reported that measures to reduce stress in broiler flocks reduced the risk of cellulitis (Bernd et al., 2020). In addition to *E*. *coli*, other bacterial species have also been reported to be associated with cellulitis, including *P*. *mirabilis*, *Aeromonas* spp., *Staphylococcus* spp., *Streptococcus* spp., and *Enterobacter* spp. (AMER et al., 2020; Sanches et al., 2020). These species seem however in our study to be less frequently associated with cellulitis.

**Table 1:** Prevalence and bacterial species isolated from 400 samples of broilers condemned with cellulitis, scratches, hepatitis, and healthy control carcasses.

Categories of condemned carcasses sampled	Lesion	Bone marrow	Meat	Prevalent bacterial species
Cellulitis (*N* = 100)	(100/100) 100%	(48/100) 48%	(12/100) 12%	*Escherichia coli* 60.9% (112\184)
				*Aeromonas* spp. 20.6% (38/184)
				*Proteus mirabilis* 12.5% (23/184)
				Staphylococcus aureus 1.6% (3/184)
				*Macrococcus caseolyticus* 1.1% (2/184)
				*Acinetobacter towneri* 1.1% (2/184)
				*Streptococcus spp*. 1.1% (2/184)
				*S*. *dysgalactiae* 0.5% (1/184)
				*Kurthia gibsonii* 0.5% (1/184)
Scratches (*N* = 100)	97%	28%	5%	*Aeromonas* spp. 37.5% (59/157)
				*E*. *coli* 29.9% (47/157)
				*P*. *mirabilis* 23.6% (37/157)
				*S*. *aureus* 3.8% (6/157)
				*Kurthia gibsonii* 1.3% (2/157)
				*Enterococcus faecalis* 1.3% (2/157)
				*Acinetobacter junii* 1.3% (2/157)
				*Micrococcus caseolyticus* 1.3% (2/157)
Hepatitis (*N* = 100)	38%	17%	2%	*E*. *coli* 47.8% (33/69)
				*Aeromonas* spp. 23.2% (16/69)
				*S*. *aureus* 11.6% (8/69)
				*P*. *mirabilis* 5.8% (4/69)
				*E*. *faecalis* 5.8% (4/69)
				*M*. *caseolyticus* 4.3% (3/69)
Hepatitis (*N* = 100)	38%	17%	2%	*Acinetobacter johnsonii* 1.4% (1/69)
Control for cellulitis	25%	7%	1%	*E*. *coli* 38.9% (23/59)
Control for scratches	25%	7%	1%	*Aeromonas* spp. 30.5% (18/59)
Control for hepatitis	23%	7%	1%	*P*. *mirabilis* 18.6% (11/59)
				*S*. *aureus* 8.5% (5/59)
				*S*. *dysgalactiae* 1.7% (1/59)
				*Enterococcus gallinarum* 1.7 (1/59)

**Figure 1:**
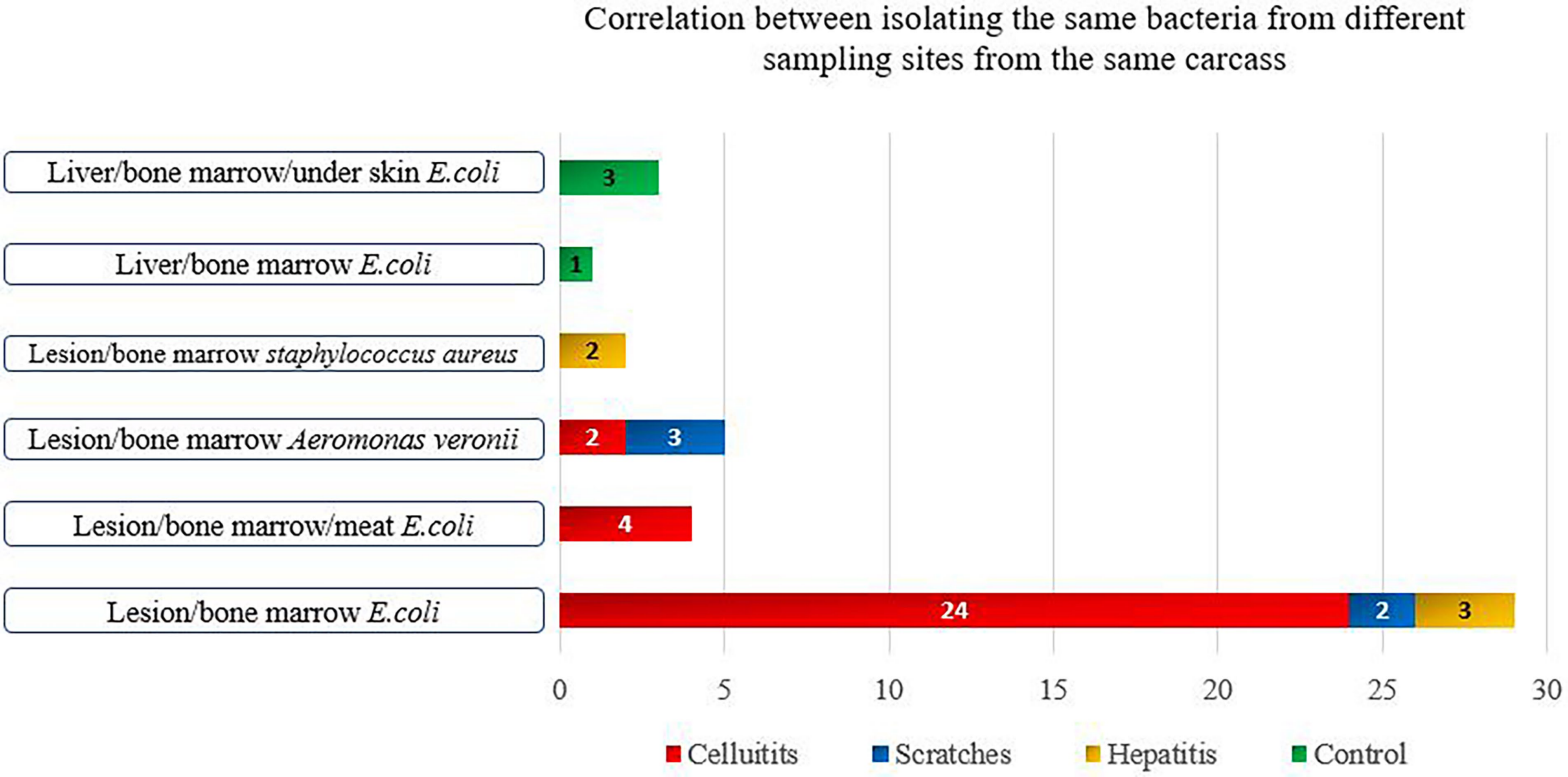
Bacterial species isolated from more than two samples’ types of the same condemned carcass.

There was a statistically significant higher occurrence of bacteria in bone marrow samples (indication of systemic infection) among carcasses with cellulitis compared to those condemned due to other causes and the control group (*p* < 0.001) (Table 2). Isolation of the same bacterial species from different sample types of the same carcass was more common among carcasses condemned with cellulitis. *E*. *coli* was detected in the sample of cellulitis, from bone marrow and meat samples of four carcasses. *A*. *veronii* was also isolated from samples of cellulitis and bone marrow in two carcasses (Figure 1). This may indicate a spread of disease, where the infection begins in the lesion before gradually spreading to become systemic and detectable in the bone marrow, and then subsequently progressing to later stages of systemic infection when the bacteria penetrate the meat. Bacteria were isolated from significantly larger proportions of bone marrow (48.0%) and meat (12.0%) samples from carcasses with cellulitis compared to other condemnation codes, indicating a higher risk of systemic infections in broilers with cellulitis, which supports the total condemnation of carcasses with cellulitis.

**Table 2:** Significance of systemic infection occurrence (isolation of bacteria from bone marrow) in the three causes of condemnation (cellulitis, scratches, and hepatitis) when compared to control samples (chi-square test).

Variables	Systemic infection				Chi-square	*P*-value
	+	-	+	-		
Cellulitis vs. control	48	52	7	93	40.1	<0.001
Scratches vs. control	28	72	7	93	13.9	<0.001
Hepatitis vs. control	17	83	7	93	3.8	0.05

#### Carcasses condemned with scratches

Among the carcass with scratches, bacteria were isolated in 97% of lesions, 28% of bone marrow, an in 5% of meat samples. *Aeromonas* spp. (37.5%) was the most frequently isolated species from all sample types, followed by *E*. *coli* (29.9%) and *P*. *mirabilis* (23.6%) (Table 1). Intact skin is the first-line defense against bacterial infections. When compromised, bacteria in the rearing house environment, including litter, can penetrate, and subsequently cause infection (Torok et al., 2009; Shepherd and Fairchild, 2010; Alfifi et al., 2020). Litter often contains *Aeromonas* spp., *E*. *coli*, and *P*. *mirabilis* which are usually present in the gut microbiota (Lu et al., 2003; Cressman et al., 2010; Chinivasagam et al., 2016; Sreeremya, 2017; Vaz et al., 2017). Thus, skin injuries allow these opportunistic bacteria to penetrate and potentially cause infections (Shepherd and Fairchild, 2010; Thøfner et al., 2019; Alfifi et al., 2020).

Systemic infections were significantly more frequent in carcasses with scratches compared to the control group (*p* < 0.001) (Table 2). Unlike for carcasses with cellulitis, it was not possible to isolate bacteria from all three sample types of any individual carcass condemned with scratches. *A*. *vironii* was isolated from both the scratch lesion and bone marrow samples in three carcasses, while *E*. *coli* was isolated from the lesion and bone marrow in two carcasses (Figure 1). Visual inspection of the scratches revealed lesions in various stages of development, ranging from minor skin injury with minimal surrounding redness to severe lesions with signs of inflammation. This large variation in severity of scratches opens up for differences in condemnation practices among meat inspectors’ which may lead to condemnation of carcasses that are safe for human consumption and ultimately more food losses. While some carcasses can be salvaged by trimming the affected areas, others cannot and should be condemned. Since scratches are the most prevalent cause for condemnation of broilers at Danish abattoirs, it is important to thoroughly investigate the predisposing management factors leading to scratches. Scratches may be prevented by progressively dimming the lights in the broiler house after 8 days of rearing and dimming the lights whenever farm workers are present (Poulsen et al., 2018). To reduce wastage and variations in how inspectors condemn carcasses with scratches, there is a need to more clearly define the different types and severity of skin lesions and to determine systemic infections in such different types of lesions.

#### Carcasses condemned with acute hepatitis

Among carcasses with acute hepatitis, the prevalence of carcasses with bacterial isolations was 38.0, 17.0, and 2.0% in the samples from the hepatitis lesion, bone marrow, and meat, respectively. The predominant bacterial species were *E*. *coli* (47.8%), *Aeromonas* spp. (23.2%) and *S*. *aureus* (11.6%) (Table 1). The common isolation of *E*. *coli* in carcasses with hepatitis corroborates previous studies (Gomis et al., 2000; Hariharan et al., 2008; Akhtar et al., 2016). *Aeromonas* spp. has also previously been isolated from liver samples of broilers with hepatitis (Dashe et al., 2013). Another study that investigated the prevalence of *Aeromonas* spp. in healthy carcasses found 66.8% of the livers from such carcasses positive for *Aeromonas* spp. (Brahmabhatt, 2011). *S*. *aureus* and *P*. *mirabilis* have also been isolated from the liver of healthy broilers (Barbour et al., 2012; Abdalrahman and Fakhr, 2015; Abolghait et al., 2020). Our findings together with the other studies reported indicate that some of the bacterial species isolated in our study from liver samples, except for *E*. *coli* and *E*. *faecalis*, may not be associated with hepatitis in poultry, indicating that these bacteria may be present in the liver but are not necessarily involved in liver inflammation. However, several Gram-positive cocci have been associated with systemic infections in broilers, i.e., septicemia including endocarditis and hepatitis. *E*. *avium*, *E*. *cecorum*, *E*. *durans*, *E*. *faecalis*, *E*. *faecium*, *and E*. *hirae* have all been associated with diseases in poultry (McNamee and Smyth, 2000; Chadfield et al., 2004; Olsen et al., 2016). *E*. *faecalis* isolates of human and poultry origin, may share identical virulence and other genes associated with pathogenesis indicating a zoonotic potential (Olsen Schønheyder et al., 2012).

There was a statistically significant difference between positive bacterial findings in samples of bone marrow among carcasses condemned with acute hepatitis compared to the control group (*p* = 0.05) (Table 2). *E*. *coli* was isolated from both hepatitis and bone marrow samples in three chickens, while *S*. *aureus* was isolated from both the hepatitis lesion and bone marrow in two carcasses (Figure 1). Overall, the lowest rates of bacterial isolation were observed in carcasses affected with hepatitis.

#### Healthy control carcasses

Bacteria were isolated from the skin (25.0%), liver (23.0%), bone marrow (7.0%), and meat (1.0%) of the healthy control carcasses. The most common species were *E*. *coli* (38.9%), *Aeromonas* spp. (30.5%), *P*. *mirabilis* (18.6%), and *S*. *aureus* (8.5%); findings that corroborate previous studies (Kitai et al., 2005; Brahmabhatt, 2011; Beninati et al., 2015; Owuna et al., 2015; Skočková et al., 2015; Bortolaia et al., 2016; Davis et al., 2018; Li et al., 2019; Sanches et al., 2019, 2021; Sarker et al., 2020; Sheir et al., 2020; Yu et al., 2021). During slaughter, carcasses are likely contaminated at various stages of processing by the microbial flora from the digestive tract, skin, and feathers, as well as by bacteria present on surfaces on processing equipment and in the chillers (Rouger et al., 2017). Carcasses may be contaminated when feathers are removed or handling of carcasses, e.g., from contaminated surfaces of conveyor belts (Arnold, 2007; Veluz et al., 2012). Fecal contamination may also occur during evisceration if the equipment is not well-adjusted and carcasses slaughtered are not of uniform size for processing. Therefore, several different bacterial species will be present on the surfaces of broiler carcasses.

The presence of bacteria under the skin of about 25.0% of the healthy control carcasses can be explained by contamination during the scalding process. The skin and feather follicles of the carcasses are often dilated during this high-temperature exposure which permits the entry of bacteria from the feathers into the skin (Rouger et al., 2017). Moreover, the skin and cuts of broiler carcasses are easily contaminated on the surface during processing due to their direct contact with contaminated equipment surfaces (Luber, 2009).

Among the healthy control carcasses, *E*. *coli* was isolated from the liver, under the skin and bone marrow of three carcasses, and the same bacterial species was isolated from the liver and bone marrow in one carcass (Figure 1). The detection of bacteria in 7% of the bone marrow samples from healthy carcasses was unexpected. However, this could be due to the early onset of systemic infection in the birds at the time of slaughter, during which the birds had not developed any gross lesions. Alternatively, it is possible that the immune systems of the birds may have overcome the infection. A previous study found that although the initial inflammatory response of the bird is sufficient to eventually control invasion and eliminate systemic and gastrointestinal infections caused by *Salmonella* spp., where the infection can persist for weeks in the bird’s gut without causing disease (Wigley, 2014). This has significant public health implications as the pathogen can typically persist long enough to be present in the carcass, particularly with broilers that are usually slaughtered at around 6 weeks of age (Wigley, 2014). It has further been reported that poultry when surviving systemic infection becomes carriers of bacteria in the bone marrow with or without visual pathological changes (Shivaprasad, 2000).

### Genetic and phenotypic analysis of *Escherichia coli*

#### Antimicrobial susceptibility analysis

MIC testing showed some resistance to ampicillin (38.6%), sulfamethoxazole (27.3%), tetracycline (27.3%), nalidixic acid (22.8%), trimethoprim (16%), ciprofloxacin (9.1%) and gentamicin (2.3%). All *E*. *coli* strains were susceptible to azithromycin, cefotaxime, ceftazidime, chloramphenicol, colistin, meropenem, and tigecycline (Table 3).

**Table 3:** Antimicrobial resistance of *E*. *coli* isolated from broiler carcasses condemned with cellulitis, scratches, and hepatitis.

Antimicrobial	No. of resistant isolates (%) (*N* = 44)
Ampicillin (AMP)	17 (38.6)
Azithromycin (AZI)	0
Cefotaxime (CTA)	0
Ceftazidime (CTZ)	0
Chloramphenicol (CHL)	0
Ciprofloxacin (CIP)	4 (9.1)
Colistin (COL)	0
Gentamicin (GEN)	1 (2.3)
Meropenem (MER)	0
Nalidixic acid (NAL)	10 (22.8)
Sulfamethoxazole (SME)	12 (27.3)
Tetracycline (TET)	12 (27.3)
Tigecycline (TIG)	0
Trimethoprim (TRI)	7 (16.0)

Whole genome sequence analysis revealed the absence of ESBL resistance genes corroborates the lack of phenotypic resistance to third-generation cephalosporins. The main beta-lactamase resistance genes detected where of the *bla*_TEM_ family including *bla*_TEM-1B_ (*n* = 12), *bla*_TEM-1C_ (*n* = 6), *bla*_TEM-220_ (*n* = 2), *bla*_TEM-106_ (*n* = 2), *bla*_TEM-135_ (*n* = 2), *bla*_TEM-126_ (*n* = 1), and *bla*_TEM-127_ (*n* = 1). The lack of ESBL resistance is likely because third-generation cephalosporins have not been used in Danish poultry production for many years (Attauabi et al., 2021).

The sulphonamide resistance gene *sul*2 (*n* = 11) was detected in 25% of the *E*. *coli* isolates all of which also showed phenotypic sulphonamide resistance. Aminoglycoside resistance genes were detected in 13.6% of the isolates, including *aph*(6)-Id (*n* = 5), *aph*(3″)-Ib (*n* = 5), *aph*(3′)-Ia (*n* = 2), *aad*A1 (*n* = 2), *aad*A5 (*n* = 1), and *aac*(3)-*Via* (*n* = 1). One isolate carrying *aad*A1 and *aac*(3)-*Via* was resistant to gentamicin. The trimethoprim resistance genes *dfr*A1 (*n* = 3), *dfr*A14 (*n* = 2), *dfr*A15 (*n* = 2), and *dfr*A17 (*n* = 1) were detected in 16% of the isolates and the tetracycline resistance genes *tet*(A) (*n* = 9) and *tet*(B) (*n* = 3) were detected in 25% of the isolates. The macrolide resistance gene *mdf*(A) was detected in all isolates, while the *sit*ABCD operon, encoding resistance to disinfectants, particularly hydrogen peroxide was detected in 77.3% of the isolates. The single *E*. *coli* isolate that harbored a *cml*A1 gene was not phenotypically resistant to chloramphenicol. Ten isolates that had a known mutation in *gyr*A (S83L), a mutation that confers resistance to quinolones were also observed to show phenotypic resistance to nalidixic acid.

Our results are consistent with those reported in the Danish Integrated Antimicrobial Resistance Monitoring and Research Programme (DANMAP) for broilers including an increasing nalidixic acid resistance and decreasing chloramphenicol and trimethoprim resistance among *E*. *coli* over the last 5 years (Attauabi et al., 2021). In general, the highest phenotypic and genotypic level of resistance with regard to sample origin was observed in isolates from scratches followed by isolates from cellulitis samples. Isolates from carcasses with hepatitis revealed the lowest level of resistance.

Overall, our results revealed lower antimicrobial resistance rates in *E*. *coli* from the condemned broilers when compared to other European countries (EFSA, 2018). This could be due to the limited and lower use of antimicrobials and effective biosecurity measures in the Danish poultry industry.

#### Genetic analyses of *Escherichia coli*

Multi-locus sequence typing (MLST) and serotyping revealed a high diversity among the *E*. *coli* isolates with 21 different sequence types (STs) and 24 different serotypes identified among the 44 *E*. *coli* sequenced isolates (Supplementary Table S1). The most frequent ST was ST117 (27.3%) which was common for *E*. *coli* isolated from cellulitis samples (45%), whereas ST48 was the second most prevalent ST (9.1%). ST69 was isolated from liver samples from condemned carcasses (hepatitis) and skin samples of carcasses condemned with scratches. Seven of the 44 isolates (16%) were serotype O78:H4 and most belonged to phylogroup G (27.3%) with only one isolate belonging to phylogroup E. In accordance with our findings, the ST117 type has previously been associated with cellulitis in Danish poultry farms (Poulsen et al., 2018). High mortalities in poultry farms in other Nordic countries were also associated with ST117 O78:H4 (Ronco et al., 2017). ST69 has been found in broilers, pork, beef, and diseased humans confirming its zoonotic potential (Manges and Johnson, 2012; Hammerum et al., 2020). The majority (75%) of isolates obtained from the lesion and bone marrow shared the same ST type and serotype.

**Figure 2:**
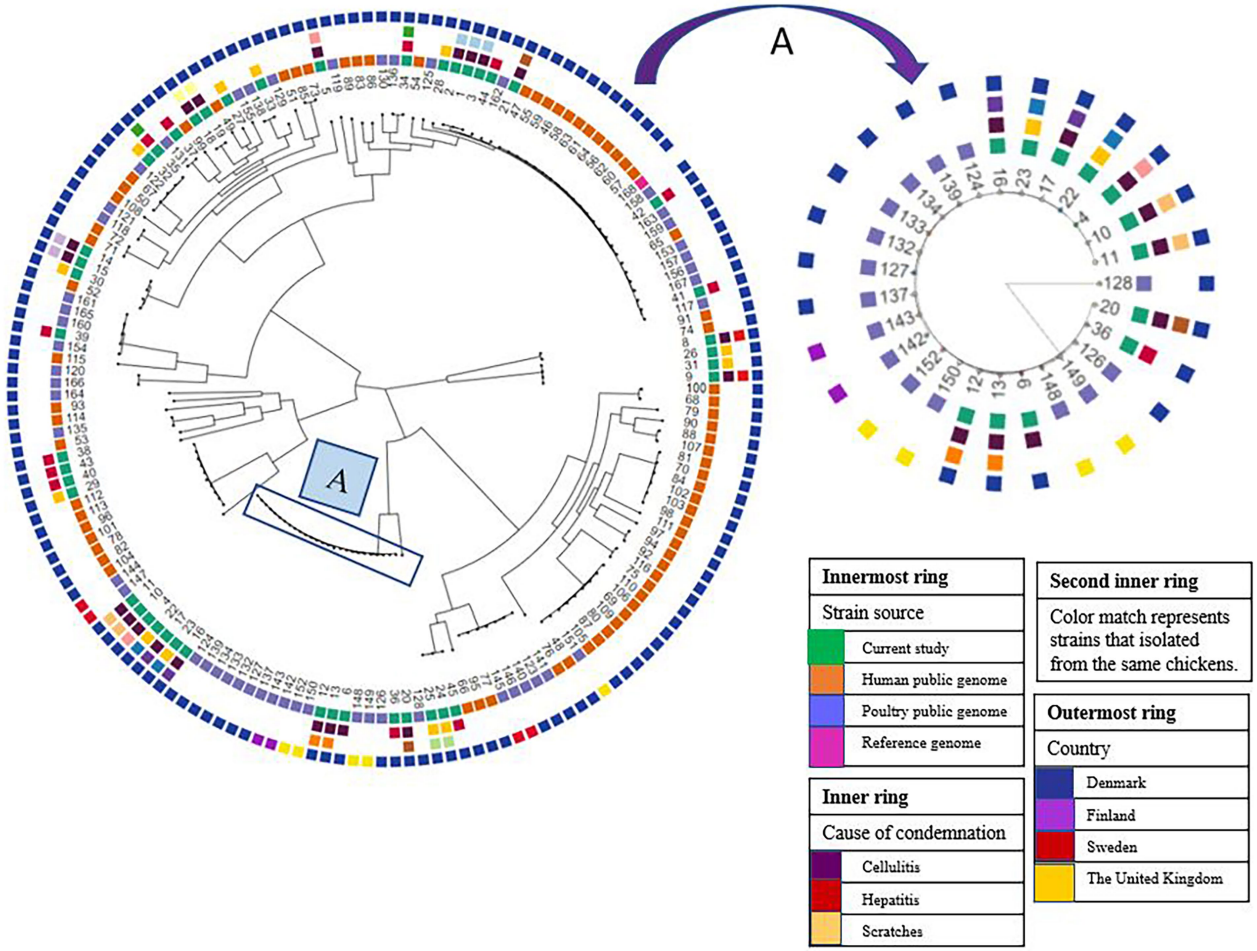
Phylogeny of *E*. *coli* isolated from broiler carcasses condemned with cellulitis, scratches, and hepatitis. Sample IDs from 1–45 represent samples from this study, whereas public genomes indicated with ID from 46–116. (A) Represents the major *E*. *coli* clade of 12 isolates (11, 10, 4, 22, 17, 23, 16, 12, 13, 6, 36, 20) all belong to ST117 mainly obtained from carcasses condemned with cellulitis. The isolates in this clade clustered together with isolates originating from poultry with cellulitis/colibacillosis in Denmark, Finland, and United Kingdom.

The ST117 O78:H4 isolates shared the following virulence genes (iroN, iss, ompT, traT, ireA, iucc, iutA, sitA, terC, ea., cleb, chuA, pic, etsc, hlyF, hra, and IpfA). The first seven genes have been reported to be associated with avian pathogenic *E*. *coli* (APEC), while the rest are mostly found in all *E*. *coli* including commensal strains. None of the ST117 O78:H4 isolates could be classified as an EXPEC or as a uropathogenic *E*. *coli* (UPEC) strain. However, ST117 O45:H4 (sample ID:36), and ST177 O161:H4 (ID:6) revealed more virulence genes known to be associated with infections in humans, and can therefore be classified as EXPEC and UPEC. Ten additional strains (ID: 8, 9, 12, 13, 24, 25, 26, 29, 31, and 45), four of them originating from cellulitis, five from scratches, and one from hepatitis could be also classified as an EXPEC and a UPEC. Three isolates were classified as an EXPEC (ID: 33, 38, and 43) including one isolate from scratches lesions and two from liver samples. Two isolates (ID: 20 and 39) from carcasses with cellulitis and hepatitis were classified as UPEC. Moreover, these EXPEC and UPEC isolates were closely related to public available clinical isolates from diseased chickens and humans as observed in the phylogenetic analysis (Figure 2). All of the isolates harbored sequences encoding from one to six plasmids. The total number of plasmid replicons was 26 different plasmid replicons. The most common detected plasmid replicons were IncFIB(AP001918) (*n* = 34), IncFII (*n* = 19), Col156 (*n* = 11), and IncFIC(FII) (*n* = 9) (Supplementary Table S1).

#### Phylogenetic analysis

Phylogenetic analysis revealed that our isolates constituted a genetically heterogeneous population with isolates distributed in five different clades. We observed a close genetic relatedness among isolates obtained from carcasses condemned with cellulitis (Figure 2). For instance, isolates 1, 2, and 3 that are genetically closely related were obtained, respectively, from meat, cellulitis lesion, and bone marrow of the same carcass. Moreover, isolates 8–9, 10–11, 12–13, 14–15, 16–17, and 18–19 collected from the cellulitis lesion and bone marrow of the same carcasses were closely related (Figure 2).

In the carcasses condemned with scratches there was also close genetic relatedness among the isolates from the different sample types obtained from the same carcass (ID: 22–23 and 24–25), i.e., isolates from meat and bone marrow of two carcasses. Of the carcasses condemned with hepatitis, only one carcass had isolates from the lesion and bone marrow (ID: 34–35), however, these two isolates displayed no genetic relatedness (Figure 2). A major clade of 12 isolates collected mainly from cellulitis samples were closely related. The isolates in this clade all belonged to ST117 with five isolates having serotype O78:H4, whereas O161:H4, O87:H4, O114:H4, and O45:H4 serotypes were shown by 3, 3, 2, 1, and 1 isolates, respectively. Isolates in this particular clade clustered together with isolates collected from poultry with cellulitis/colibacillosis originating from Denmark, Finland, and the United Kingdom (Figure 2). Similarly, it has been reported that *E*. *coli* ST117 O78:H4 clones appeared to be circulating among broilers in poultry farms in the Nordic countries. It was concluded that the spread of this clone was vertically through grandparents of broilers that were reared in Sweden and then imported and spread at poultry farms importing day-old chickens from Sweden (Ronco et al., 2017). Since isolates from carcasses with cellulitis reported in the United Kingdom clustered with the isolates from broilers in Denmark and Finland, we could speculate that the original source of spread of this clone was not from Sweden, but from the United Kingdom as Swedish grandparents are primarily imported from Scotland (Ronco et al., 2017).

There was a close relationship among the *E*. *coli* isolates that were obtained from bone marrow and samples of cellulitis and meat of the same carcass. Furthermore, the WGS analysis confirmed the presence of known virulence genes which indicates that the *E*. *coli* strains are pathogenic. Our findings thus support the hypothesis that the *E*. *coli* initially was associated with the development of cellulitis, and then spread to cause systemic infection. Four *E*. *coli* isolates from lesions and bone marrow of two carcasses condemned with scratches were also closely genetically related. However, follow-up studies are needed to confirm if *E*. *coli* from samples of scratches cases are able to develop into systemic infections.

## Conclusion

Overall, a total of 469 bacterial isolates were recovered from different sample types of the 400 carcasses collected. Bacteria were more frequently isolated from carcasses condemned with cellulitis (184/469) as compared with carcasses condemned with hepatitis (69/469). *E*. *coli* was the main isolated bacterial species from all sample types from condemned carcasses, indicating that infection caused by *E*. *coli* is the main cause of carcass condemnation in Danish broilers. A clonal relationship was found between *E*. *coli* isolated from different sample types of the same carcass condemned with cellulitis and scratches. This suggests that an infection may be initiated in the tissue of the lesion, and then gradually develop into a systemic infection which can be detected through the isolation of bacteria in the bone marrow and muscle (meat) samples. A dominant clade including 12 isolates was found to be closely related and primarily associated with systemic infection. Isolates from this clade belonged to ST117 and clustered together with strains isolated from diseased and condemned broiler carcasses from Scandinavian countries and United Kingdom. Further research is needed to assess risk factors and how to control the transmission of this clade.

## Data availability statement

The datasets presented in this study can be found in online repositories. The names of the repository/repositories and accession number(s) can be found in the article/Supplementary material.

## Author contributions

AA, AD, JC, and MS designed the study. AA collected all samples, performed various laboratory analyses, and wrote the first draft of the manuscript. JC and YH provided guidance on laboratory analysis. AA and YH performed bioinformatic analysis. AD supervised the study. All authors revised the manuscript and approved the final version.

## Funding

This work was supported by King Faisal University, Ministry of Education Kingdom of Saudi Arabia which provided the financial cost of the research and a Ph.D. scholarship for AA, project number: (1160961001), 50-KU (Copenhagen University), (30368200) Section of Food Safety and Zoonoses.

## Conflict of interest

The authors declare that the research was conducted in the absence of any commercial or financial relationships that could be construed as a potential conflict of interest.

## Publisher’s note

All claims expressed in this article are solely those of the authors and do not necessarily represent those of their affiliated organizations, or those of the publisher, the editors and the reviewers. Any product that may be evaluated in this article, or claim that may be made by its manufacturer, is not guaranteed or endorsed by the publisher.

## Footnotes

^1^ http://www.eucast.org

^2^ https://www.genomicepidemiology.org/services/

^3^ https://github.com/tseemann/snippy
